# Triptolide Transcriptionally Represses HER2 in Ovarian Cancer Cells by Targeting NF-**κ**B

**DOI:** 10.1155/2012/350239

**Published:** 2012-12-24

**Authors:** Chien-Chih Ou, Yuan-Wu Chen, Shih-Chung Hsu, Huey-Kang Sytwu, Shih-Hurng Loh, Jhy-Wei Li, Jah-Yao Liu

**Affiliations:** ^1^Department of Obstetrics and Gynecology, Tri-Service General Hospital, No. 325, Section 2, Cheng-Kung Road, Neihu, Taipei 11490, Taiwan; ^2^School of Dentistry, National Defense Medical Center, No. 161, Section 6, Minquan E. Road, Neihu, Taipei 11490, Taiwan; ^3^Department of Oral and Maxillofacial Surgery, Tri-Service General Hospital, Taipei 11490, Taiwan; ^4^Kang-Ning Junior College of Medical Care and Management, Taipei 11490, Taiwan; ^5^Department of Microbiology and Immunology, National Defense Medical Center, Taipei 11490, Taiwan; ^6^Department of Pharmacology, National Defense Medical Center, Taipei 11490, Taiwan; ^7^Department of Pathology, Da-Chien General Hospital, Miaoli 36052, Taiwan; ^8^Department of Nursing, Jente Junior College of Medicine, Nursing, and Management, Miaoli 35664, Taiwan

## Abstract

Triptolide (TPL) inhibits the proliferation of a variety of cancer cells and has been proposed as an effective anticancer agent. In this study, we demonstrate that TPL downregulates HER2 protein expression in oral, ovarian, and breast cancer cells. It suppresses HER2 protein expression in a dose- and time-dependent manner. Transrepression of HER2 promoter activity by TPL is also observed. The interacting site of TPL on the HER2 promoter region is located between −207 and −103 bps, which includes a putative binding site for the transcription factor NF-**κ**B. Previous reports demonstrated that TPL suppresses NF-**κ**B expression. We demonstrate that overexpression of NF-**κ**B rescues TPL-mediated suppression of HER2 promoter activity and protein expression in NIH3T3 cells and ovarian cancer cells, respectively. In addition, TPL downregulates the activated (phosphorylated) forms of HER2, phosphoinositide-3 kinase (PI3K), and serine/threonine-specific protein kinase (Akt). TPL also inhibits tumor growth in a mouse model. Furthermore, TPL suppresses HER2 and Ki-67 expression in xenografted tumors based on an immunohistochemistry (IHC) assay. These findings suggest that TPL transrepresses HER2 and suppresses the downstream PI3K/Akt-signaling pathway. Our study reveals that TPL can inhibit tumor growth and thereby may serve as a potential chemotherapeutic agent.

## 1. Introduction

Triptolide (TPL) is a diterpenoid triepoxide derived from the herb *Tripterygium wilfordii *[1]. TPL has been demonstrated to possess a unique bioactive spectrum of anti-inflammatory, immunosuppressive, antifertility, anticystogenesis, and anticancer activities [2]. Recently, it was found that TPL reduces proliferation and induces apoptosis in cancer cells [2–4]. Furthermore, it inhibits tumor growth and metastases in mouse xenografts of melanoma, breast cancer, gastric carcinoma, bladder cancer [3], and pancreatic cancer [4]. Previously, we found that TPL has a strong antitumor effect against oral cancer cells through caspase-mediated apoptosis [5]. In addition, TPL circumvents drug resistance and enhances the antitumor effect of 5-fluorouracil [6]. The understanding of the molecular mechanisms by which TPL inhibits cancer cell growth may shed light on cancer therapy. 

The HER2 gene, also known as *neu* (in mouse) or* erbB2*, encodes a 185-kDa transmembrane receptor tyrosine kinase and is a member of the epidermal growth factor receptor family [7]. Overexpression of HER2 is found in ~30% of human breast cancers and in many other cancer types [8]. HER2 phosphorylates downstream substrates and activates a variety of signaling cascades, including the phosphatidylinositol-3 kinase (PI3K), serine/threonine-specific protein kinase (Akt), and Ras/mitogen-activated protein kinase (MAPK) pathways. These regulatory signaling cascades promote cell survival, tumor growth, and metastasis [9, 10].

 TPL reduces PI3K activity, which is a downstream of HER2 signaling pathway. More importantly, TPL blocks the activity of NF-*κ*B, which activates HER2 gene transcription [11, 12]. Thus, we hypothesized that TPL-induced tumor regression is attributed to its repression on HER2 activity. The molecular mechanism by which TPL acts on the HER2 signaling cascade is investigated.

## 2. Materials and Methods

### 2.1. Cell Culture and Plasmids

KB (ATCC) and OEC-M1 (a cell line derived from gingival epidermal carcinoma, an indigenous oral cancer cell line in Taiwan) [5] human oral cancer cell lines were maintained in RPMI 1640 medium supplemented with 10% fetal bovine serum (FBS, HyClone and Logan, UT). SKOV-3 (Bioresource Collection and Research Center, BCRC), OVCAR-3 (BCRC), and TOV-21G (BCRC) human ovarian cancer cell lines were grown in McCoy's 5A, RPMI 1640 (Gibco BRL, Gaithersburg, MD), and MCDB 105/Medium199 (Sigma-Aldrich, St. Louis, MO) media, respectively, supplemented with 10–20% FBS (HyClone). BT-474 (ATCC), MCF-7/HER (stably HER2-transfected MCF-7), and MCF-7 (ATCC) human breast cancer cell lines were grown in Dulbecco's Modified Eagle's Medium (DMEM)/F12 medium supplemented with 10% FBS. NIH3T3 (BCRC) mouse fibroblasts were maintained in DMEM high glucose supplemented with 10% FBS. Cells were grown at 37°C in a humidified incubator with 5% CO_2_. The NF-*κ*B (p65) and NF-*κ*B (p50) expression vectors were gifts from Dr. S. M. Huang (Department of Biochemistry, Taipei 114, Taiwan).

### 2.2. Cell Transfection

A liposome-mediated transfection method was used to transfect cells. Briefly, plasmids were diluted in Opti-MEM I (Gibco BRL) medium and then mixed with Lipofectamine 2000 (Gibco BRL) at room temperature for 20 min. The lipoplex complex was added to the cells for 6 h, and the cells were refreshed with complete medium.

### 2.3. Western Blot Analysis

Western blot analysis was conducted as described by Ou et al. [13]. The membranes were then incubated with the primary antineu antibody (Ab-3; Oncogene Science, Cambridge, MA), antiphospho-neu (Tyr1248) antibody (Ab-18; NeoMarkers, CA), anti-Akt antibody (Cell Signaling, Beverly, MA), antiphospho-Akt (Ser473) antibody (Cell Signaling), anti-PI3K antibody (Cell Signaling), antiphospho-(Tyr) p85 PI3K antibody (Cell Signaling), anti-NF-*κ*B (p65) antibody (Epitomics, Burlingame, CA), anti-NF-*κ*B (p50) antibody (Epitomics), and antiactin (Chemicon, San Diego, CA) antibody in fresh 5% skim milk-Tris-buffered saline Tween-20 buffer for 1 h at room temperature or 4°C overnight. The membranes were washed and incubated with horseradish peroxidase-conjugated goat antimouse or goat antirabbit IgG secondary antibody (Santa Cruz Biotechnology, CA). Visualization was performed with an Amersham-enhanced chemiluminescence system. Responsive bands were quantified by Adobe Photoshop software. All experiments were repeated three times.

### 2.4. Cytotoxicity and Viability Analysis by MTT Assay

To evaluate growth inhibition, the 3-(4,5-dimethylthiazol-2-yl)-2,5-diphenyltetrazolium bromide (MTT) metabolic assay was performed [14]. Briefly, cells were seeded into 96-well microtiter plates and incubated overnight in 180 *μ*L media containing 10% FBS. For the drug sensitivity assay, cells were treated with TPL at indicated concentrations for 72 h. After treatment, the viable cells were stained with 20 *μ*L of MTT dye (5 mg/mL in PBS; Sigma-Aldrich) for 4 h, and then 170 *μ*L medium was removed. The MTT formazan formed by metabolically viable cells was dissolved in 200 *μ*L DMSO, and the absorbance was read at 545 nm with a reference at 690 nm (Emax, Molecular Device Inc.). All experiments were performed in triplicate and reproduced at least three times.

### 2.5. Quantitative Real-Time Reverse-Transcription PCR (RT-qPCR)

Total RNA was isolated using the Trizol method (Invitrogen, Carlsbad, CA), with homogenization of the cancer cells in Trizol lysis buffer followed by chloroform extraction (Life Technologies). For cDNA synthesis, 5 *μ*g total RNA was reverse transcribed at 50°C for 60 min using 200 units Superscript III Reverse Transcriptase (Invitrogen). Primer pairs for HER2 (sense 5′-CCC AAC CAG GCG CAG AT-3′; antisense 5′-AGG GAT CCA GAT GCC CTT GTA-3′), were used for gene amplification. The SYBR Green Master Mix Kit (Bio-Rad, Hercules, CA) was used for all qPCRs. Briefly, PCR was performed as follows: 94°C for 2 min, followed by 40 cycles of denaturation at 94°C for 15 s, annealing at 61.4°C for 30 s, extension at 72°C for 45 s, and a final extension at 72°C for 10 min. Data were collected in triplicate and normalized to GAPDH expression.

### 2.6. Construction of HER2 Promoter Deletion Plasmids

Genomic DNA was isolated from SKOV-3 cells. To construct HER2 reporter deletion vectors, PCR was used to introduce *Hin*dIII and *Xho*I restriction enzyme sites at the 5′- and 3′-ends of the promoter sequence of the human HER2 gene with four forward primers F1, F2, F3, and F4 and reverse primer R1 (at −103). These PCRs induced promoter deletions at −1067, −871, −495, and −207, respectively. The promoter fragments were cloned into the pGL4 vector (Promega) and sequenced, resulting in the promoter reporter vectors pGL4-HER2-F1-Luc, pGL4-HER2-F2-Luc, pGL4-HER2-F3-Luc, and pGL4-HER2-F4-Luc, respectively.

### 2.7. HER2 Promoter Activity Assay

The HER2 promoter luciferase assay was described previously [13]. Briefly, cells were cotransfected with pNeulit (containing luciferase gene driven by *neu* promoter) or HER2 promoter deletion constructs and pCMV-*β*-gal (as a reference control for transfection efficiency). Cell lysates were prepared before the luciferase activity assay by following the manufacturer's (Promega) instructions. The measured luciferase activity levels were normalized against the *β*-galactosidase (*β*-gal) standard. These experiments were repeated at least four times.

### 2.8. Xenograft Tumor Model

This study was carried out in strict accordance with the recommendations in the Guide for the Care and Use of Laboratory Animals of the National Defense Medical Center (NDMC). The protocol was approved by the Committee on the Ethics of Animal Experiments of NDMC. Eight-week-old BALB/cAnN.Cg-*Foxn*1^*nu*^/CrlNarl (National Laboratory Animal Center, Taiwan) mice were maintained in microisolators under specific pathogen-free conditions. These mice were fed sterile food and chlorinated sterile water. Eight mice were divided into two groups; each group of mice was subcutaneously injected with 5 × 10^6^ SKOV-3 cells. Four mice were further treated with TPL (0.15 mg/kg BW/day/i.p.), and another four mice were injected daily with a vehicle PBS control. TPL was first injected on day 0 and continuously administered until day 18 in SKOV-3-bearing and control mice, respectively. The size of the transplanted tumors and mice body weight were measured by gauged calipers every 3 days, and the tumor volume was calculated using the formula: volume (*V*) = 1/2 × (length × width^2^). At the end of treatment, the mice were sacrificed, and the tumors were removed, weighed, and photographed.

### 2.9. Immunohistochemical (IHC) Stain and Quantification

Immunohistochemical staining was done using the avidin-biotin method as described previously [15]. For quantitative evaluation of HER2 expression, at least three representative high-power fields (1000x) of each tumor were immunostained with HER2 monoclonal antibody (Dako, Osaka, Japan) and examined under an Olympus BX-50 microscope (Olympus Optical, Tokyo, Japan) with a photo-fixed halogen light source. A digital camera (Olympus C-2020Z) transmitted high-quality images into a computer. Manual mode was used to ensure that the image intensity was kept at a constant optimal level. Three ordered categories of HER2 expression were then scored 1–3, followed by the commercial HercepTest scoring system [15]. The percentage of Ki-67-positive (Dako, Copenhagen, Denmark) cells was determined in each case by counting cells in three contiguous high-power fields using a cell counter until 1000 cells were assessed.

### 2.10. Statistical Analysis

The results are expressed as the mean ± SEM. A *t* test was used to determine whether any significant relationships existed among quantitative results. *P* values <0.05 were considered significant. *P < 0.05; **P < 0.01.

## 3. Results

### 3.1. Downregulation of HER2 by TPL

**Figure 1:**
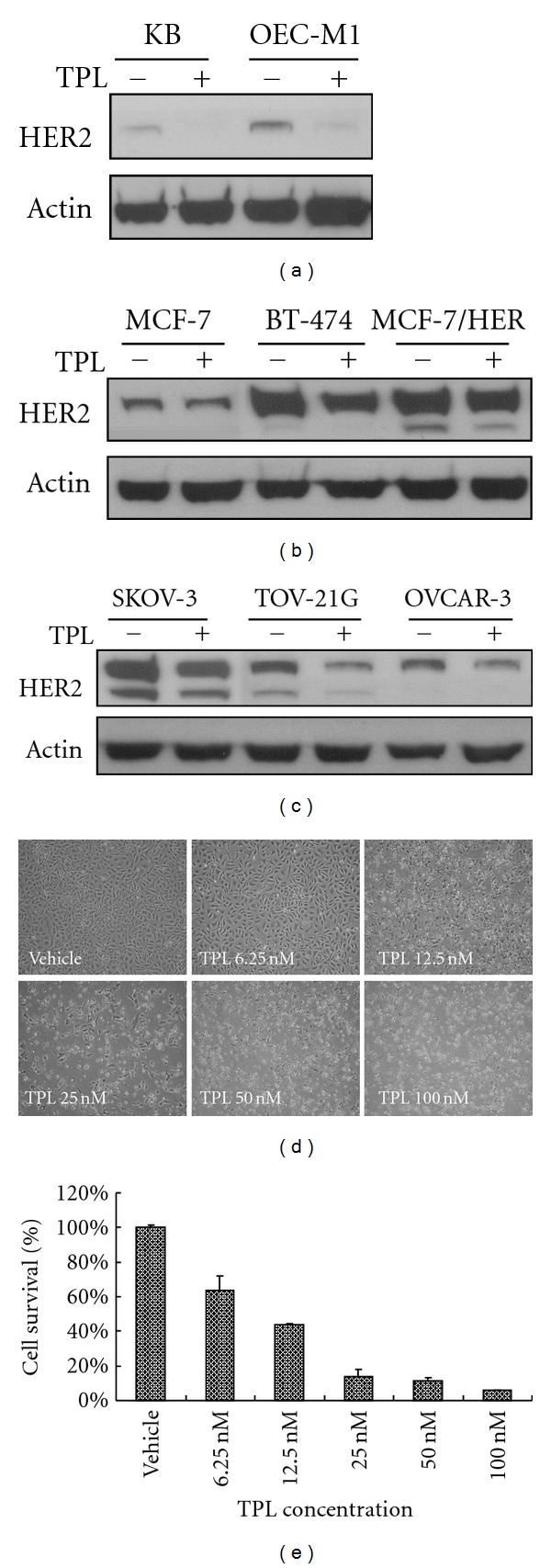
Downregulation of HER2 expression by TPL in oral, breast, and ovarian cancer cell lines. (a) KB and OEC-M1 human oral cancer cell lines; (b) BT-474, MCF-7/HER, and MCF-7 human breast cancer cell lines; and (c) SKOV-3, OVCAR-3, and TOV-21G human ovarian cancer cell lines were treated with 50 nM of TPL or vehicle (0.01% DMSO) control for 24 h. Cell lysates were immunoblotted with antibodies specific for HER2/neu or actin. SKOV-3 cells were treated with the indicated dose of TPL for 72 h. Morphological variations were recorded (d), and growth inhibition by TPL was quantified (e). The experiment was repeated three times.

**Figure 2:**
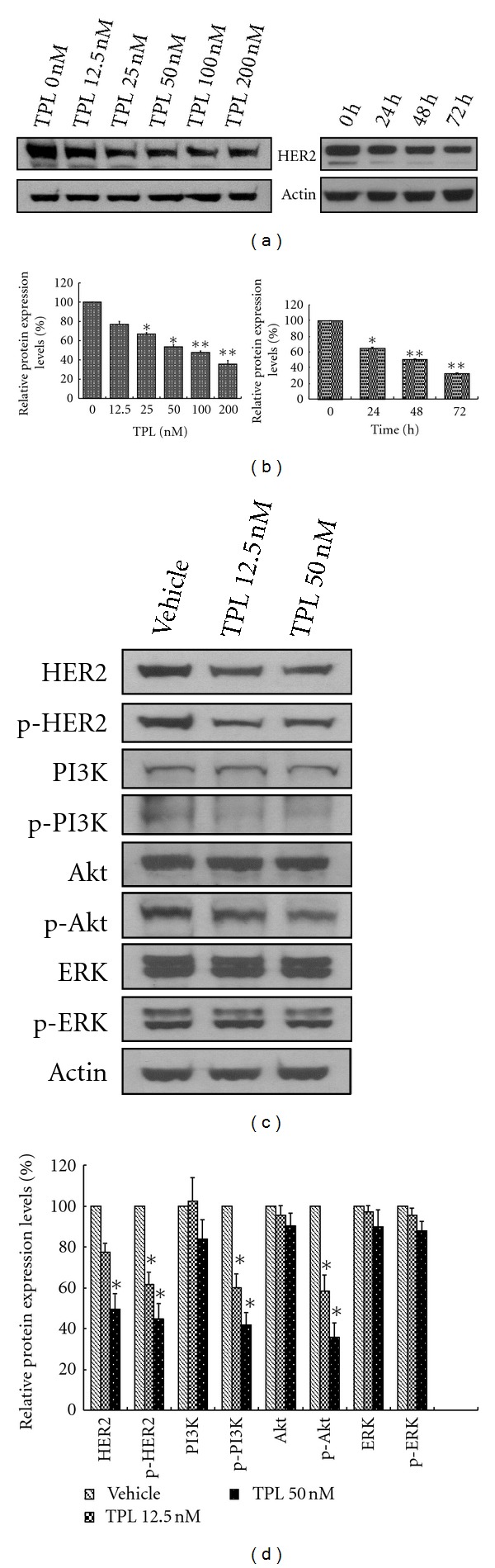
TPL downregulates HER2 expression and PI3K/Akt activity in SKOV-3 cells. (a) SKOV-3 cells were treated with various doses of TPL or PBS for 48 h (left panel) and 50 nM of TPL for the indicated time (right panel). HER2 and actin protein levels were determined by western blotting. Densitometric analysis of the percentage of decrease was determined using Adobe Photoshop software (b). HER2 expression was downregulated by TPL in SKOV-3 cell line in a dose- and time-dependent manner. (c) Downregulation of HER2 and phospho-PI3K/Akt by TPL. SKOV-3 cells were treated with the indicated concentration of TPL for 48 h. Western blot analysis of cell lysates was performed with antibodies recognizing HER2, phospho-HER2-Y1248 (p-HER2), p85 PI3K, phospho-Y p85 PI3K (p-PI3K), Akt, phospho-Akt-S473 (p-Akt), extracellular signal-regulated kinase (ERK), phospho-extracellular signal-regulated kinase-T202/Y204 (p-ERK), and actin. The densitometric analysis was performed as previously mentioned and is shown in (d). The data are presented as the mean ± standard error from three independent experiments. *P < 0.05 and **P < 0.01 versus the vehicle-treated control group.

As TPL reduces PI3K activity [16], we investigate if TPL downregulates the expression of HER2, which is upstream of PI3K activity, in cancer cells. As shown in Figures 1(a)–1(c), TPL suppresses HER2 expression in the KB and OEC-M1 oral cancer cell lines; the MCF-7, BT-474, and MCF-7/HER breast cancer cell lines; and the SKOV-3, TOV-21G, and OVCAR-3 ovarian cancer cell lines. Because SKOV-3 ovarian cancer cells overexpress HER2 [13], SKOV-3 cells were used to explore the mechanism by which TPL suppresses HER2. We demonstrated that TPL inhibits SKOV-3 cell proliferation in vitro at 72 h in a dose-dependent manner (Figure 1(d)). The quantification of these results is shown in Figure 1(e). Moreover, western blot analysis revealed that TPL decreases HER2 expression in SKOV-3 cells in a dose- and time-dependent manner (Figures 2(a) and 2(b)). As mentioned above, our results reveal that TPL is an effective antitumor agent not only in ovarian cancer cells but also in oral and breast cancer cells.

### 3.2. Downregulation of PI3K/Akt Signaling by TPL

The HER2-stimulated intracellular signaling pathways include the Ras/ERK and PI3K/Akt cascades. Because TPL suppresses HER2 protein expression, we examines if TPL interferes with HER2 and affects these two pathways in ovarian cancer cells. As demonstrated in Figure 2(c), TPL significantly suppresses both total and phosphorylated (activated) HER2 expression in SKOV-3 cells (*P* < 0.05) after 24 h. However, total ERK and phospho-ERK (activated form of ERK) are not altered by TPL treatment (Figure 2(d)). We then investigated if TPL affects the PI3K/Akt signaling cascade. After 24 h of TPL treatment, phospho-PI3K and phospho-Akt are both dramatically downregulated (Figures 2(c) and 2(d)). More than 50% inhibition by TPL is observed at a concentration of 50 nM (Figure 2(d)). At the same time, PI3K and Akt total protein levels remain unchanged (Figures 2(c) and 2(d)). In summary, these data demonstrate that TPL exerts inhibitory effects on HER2 and the PI3K/Akt signaling pathway.

### 3.3. TPL Represses HER2 Gene Activity

**Figure 3:**
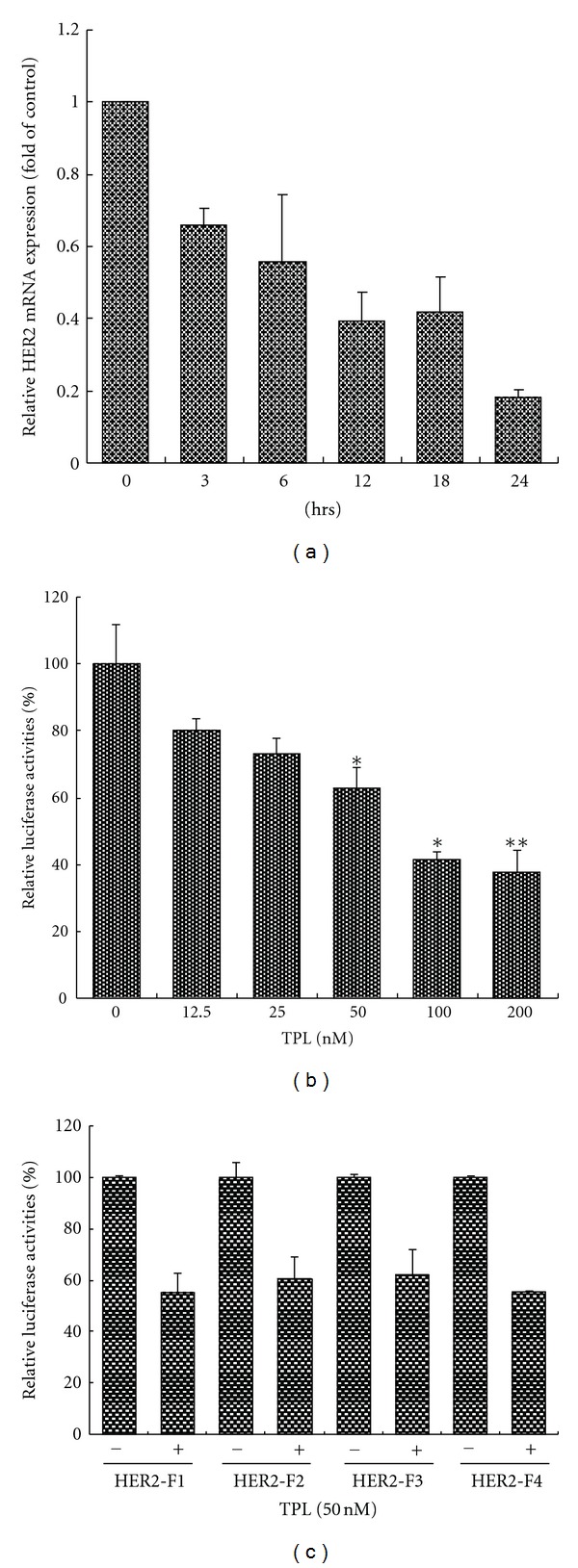
Transcriptional repression of HER2 by TPL. (a) SKOV-3 cells were treated with 50 nM of TPL and harvested at the indicated time. The quantitative PCR was performed as described in Section 2. (b) TPL inhibits *neu* promoter activity. For the luciferase assay, NIH3T3 cells were cotransfected with *neu* promoter luciferase (0.8 *μ*g) and pCMV-*β*-gal (0.2 *μ*g) plasmid DNA for 6 h and then treated with various concentrations of TPL for 24 h. The activity of luciferase in relative light units (RLUs) was normalized against *β*-gal activity. (c) The pGL4-HER2-F1-Luc through pGL4-HER2-F4-Luc constructs were transfected into NIH3T3 cells for 6 h, and then 50 nM TPL was added for 24 h. The RLUs were determined as above. TPL downregulated reporter expression at the HER2 F4 (from −207 to −103) region.

To elucidate the mechanisms by which TPL downregulates HER2, we assayed HER2 mRNA level over a treatment time course. Consistent with the HER2 protein level, RT-qPCR analysis revealed that HER2 mRNA is significantly downregulated by TPL after 3 hours of treatment (Figure 3(a)). Only about 20% of HER2 mRNA remains after 24 h. The transcriptional activity of the HER2 promoter is also analyzed. In the transient expression assays, using NIH3T3 (Figure 3(b)) as recipient cells, cotransfected with pNeulit and pCMV-*β*-gal, TPL treatment causes a significant decrease in *neu* promoter luciferase activity in a dose-dependent manner. Because pNeulit is a mouse *neu* promoter construct, we cloned the human HER2 promoter which was subsequently divided it into four fragments, F1–F4, containing bp from −1067 to −103, from −871 to −103, from −495 to −103, and from −207 to −103, respectively. These fragments were then subcloned into the luciferase reporter as described in Section 2, and the transcriptional activity of the HER2 reporter constructs was monitored following a transient transfection in NIH3T3 cells. The results shown in Figure 3(c) demonstrate that about 40% of all four promoter activities are suppressed by TPL. Thus, the F4 region may be the target site for TPL. Through bioinformatics research, we identified a putative NF-*κ*B binding site within this region.

### 3.4. Overexpression of NF-*κ*B Prevents the TPL-Induced Decrease in HER2 Protein

**Figure 4:**
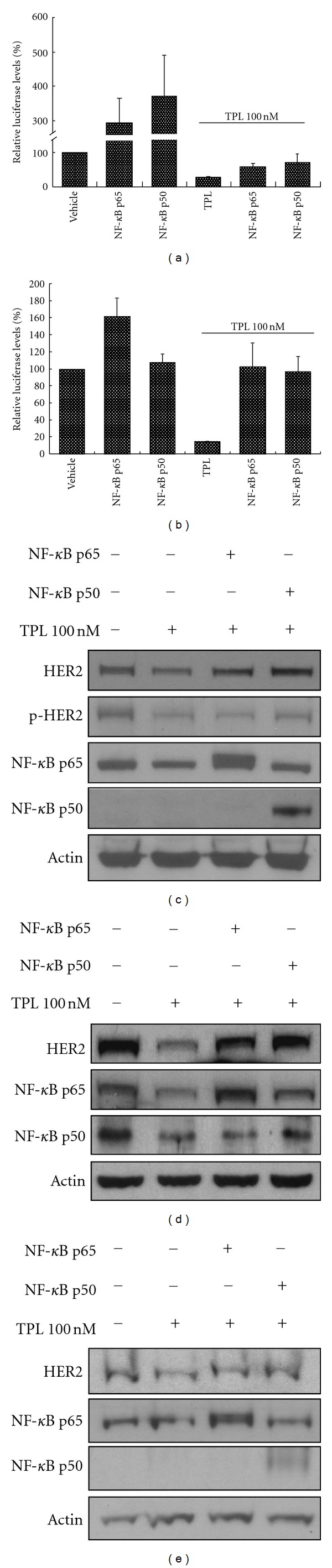
NF-*κ*B prevents the reduction of HER2 expression caused by treatment with TPL. NIH3T3 cells were transfected with NF-*κ*B-p65 or -p50 (2 *μ*g) or vector only. After 6 h, TPL (100 nM) or vehicle was added to the medium for 24 h. Luciferase assays were performed to detect HER2-F1 (a) and HER2-F4 (b) promoter activities. (c) SKOV-3 (d), TOV-21G (e), and OVCAR-3 cells were transfected with NF-*κ*B-p65 or -p50 (2 *μ*g) or vector only for 24 h and then treated with TPL or vehicle for another 24 h. Cell lysates were immunoblotted with antibodies specific for HER2/neu, p-HER2, NF-*κ*B-p65, NF-*κ*B-p50, or actin.

NF-*κ*B is a protein complex that includes the p65 and p50 subunits and modulates the transcription of several genes [17]. We found that HER2 promoter activity is enormously enhanced in NIH3T3 cells after transient cotransfection of the constructs containing the p65 or p50 subunits and the pGL4-HER2-F1-Luc plasmid (Figure 4(a)). Both NF-*κ*B-p65 and NF-*κ*B-p50 constructs prevent the TPL-induced reduction of HER2 promoter activity in NIH3T3 cells (Figure 4(a)). Moreover, NF-*κ*B restores the HER2-F4 promoter activity that is repressed by TPL in NIH3T3 cells (Figure 4(b)). We also assayed the protein levels of HER2 and NF-*κ*B in TPL-treated NF-*κ*B-overexpressing SKOV-3, TOV-21G, and OVCAR-3 cells. As shown in Figure 4(c), TPL downregulates both endogenous HER2 and NF-*κ*B-p65 protein expression. In addition, both NF-*κ*B-p65 and NF-*κ*B-p50 constructs prevent the TPL-induced reduction of HER2 protein level in SKOV-3 cells, which have no endogenous NF-*κ*B-p50. Similar results were also found in TOV-21G and OVCAR-3 cells (Figures 4(d) and 4(e)).

### 3.5. TPL Suppresses the Expression of HER2 and Ki-67 In Vivo

**Figure 5:**
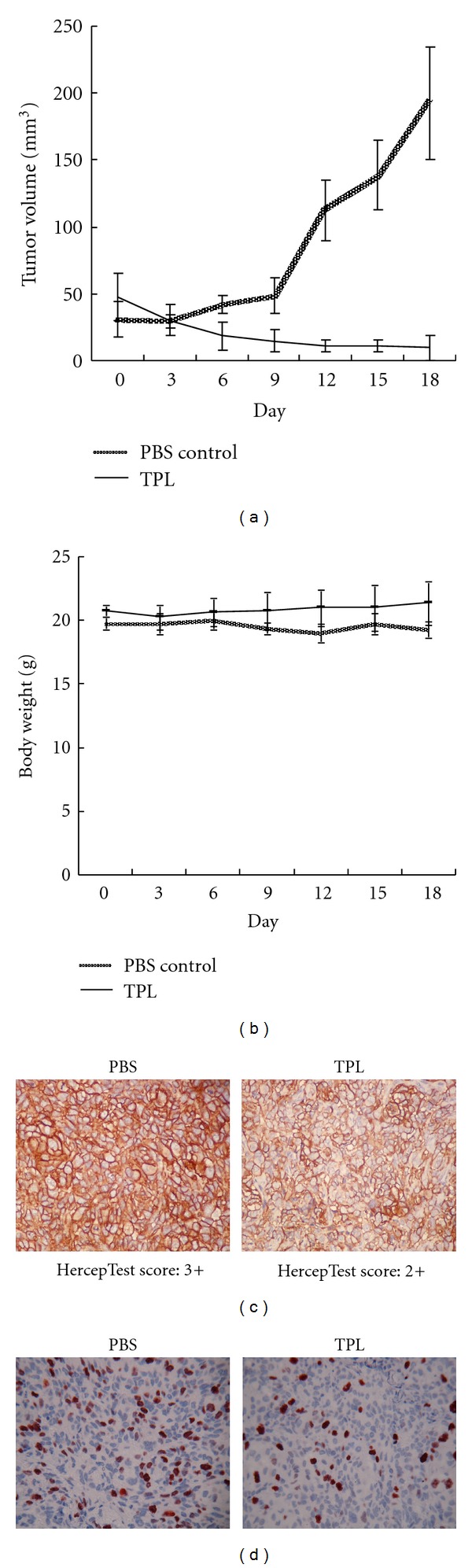
A nude mouse model showing inhibition of SKOV-3 xenograft tumor growth as well as HER2 and Ki-67 expression by TPL. (a) Daily treatment with TPL (0.15 mg/kg) after SKOV-3 tumor xenograft transplantation significantly reduced tumor size compared with vehicle. (b) Body weight in mice was not significantly different between the TPL-treated and PBS-treated groups. (c) Downregulation of HER2 and (d) Ki-67 expression by TPL in SKOV-3-induced xenograft solid tumors in nude mice. The IHC analyses of SKOV-3-xenografted tumors were taken from the inoculated nude mice. TPL significantly reduced HER2 expression (brown color) in tumor sections from HercepTest scores of 3+ to 2+ (*n* = 5) (c). In addition, TPL-treated mice had significantly less Ki-67 protein (red color) than vehicle controls (d) (*n* = 4).

To examine whether TPL inhibits the growth of HER2-overexpressing tumor cells in vivo, tumorigenicity test is performed in athymic nude mice growing SKOV-3 tumor. The mean tumor volumes of nude mice treated with TPL are significantly smaller than those treated with PBS (Figure 5(a)). These data demonstrate that TPL significantly inhibits the proliferation of HER2-overexpressing tumor cells in nude mouse xenografts. The body weights of these TPL-treated mice were not significantly changed (Figure 5(b)). Furthermore, we also tested whether TPL inhibits HER2 protein and Ki-67 expression (a proliferation marker) in nude mice. Tumor sections were immunostained for HER2 and Ki-67. We applied the semiquantitative HercepTest scoring system to measure the expression of HER2 [15]. The HercepTest score in the PBS control and TPL-treated groups were 3+ and 2+, respectively (Figure 5(c)). In an in vitro study, TPL suppressed Akt activity, which is a key mediator of antiapoptosis and cell proliferation. We also examined the Ki-67 proliferation index in these tumor sections. As shown in Figure 5(d), the percentage of cells with a positive Ki-67 expression in the group treated with TPL is significantly lower than the PBS-treated control group (16.7% versus 26.5%). These immunohistochemistry data are consistent with the protein levels reported above (Figures 1 and 2). All together, these data suggest that TPL significantly inhibits HER2 and Ki-67 expression in vivo.

## 4. Discussion

TPL, a pure compound extracted from the traditional Chinese medicinal plant* T. wilfordii*, has significant cytotoxic effects on different types of tumors. The mechanisms of action of TPL, for a long time, were hypothesized to specifically target some transcription factors. It elicits inhibitory effects upon nuclear factor-*κ*B (NF-*κ*B), heat shock transcription factor1 (HSF1), activator protein-1 (AP-1), nuclear factor of activated T-cells (NF-AT), and hypoxia-induced factor-1*α* (HIF-1*α*), while it has no impact on cytomegalovirus-luciferase or SV40-luciferase reporters [2]. In this study, we demonstrated that TPL inhibits the proliferation of HER2-overexpressing human ovarian cancer SKOV-3 cells. Further analysis indicated that TPL effectively inhibits HER2 expression in oral, breast, and ovarian cancer cell lines (Figures 1 and 2). In addition, HER2 promoter activity and mRNA level are reduced by TPL (Figures 3(a) and 3(b)), suggesting that TPL downregulates HER2 expression by diminishing HER2 gene transcription. In addition, recently, TPL is demonstrated to cause transcriptional inhibition, initially believed to target specific transcription factors but recently revealed to cause global transcription inhibition via the largest subunit of RNA polymerase II (RPB1). Two pathways are proposed that TPL interacts with XPB and CDK7 to cause transcriptional inhibition. TPL binds to human XPB, a subunit of the transcription factor TFIIH, and inhibits its DNA-dependent ATPase activity, which leads to the inhibition of RNA polymerase II-(RNAPII-)mediated transcription [2, 18]. In addition, TPL also triggers CDK7 to phosphorylate Rpb1 and induces degradation of RNAPII [19]. Thus, the downregulation of HER2 and NF-*κ*B gene expression by TPL may be also due to its effects on RNAPII.

 In promoter deletion assays, because the transient transfection rate of SKOV-3 cells is low [13], NIH3T3 cells were used as recipient cells to measure luciferase activity. We found that TPL-mediated reduction of HER2 promoter activity is specifically prevented by NF-*κ*B (Figures 4(a) and 4(b)). By comparing the results of Figures 4(a) and 4(b), we discovered that the F4 fragment contains a binding site for NF-*κ*B-p65 but not NF-*κ*B-p50. In addition, Figure 4 also reveals that NF-*κ*B-p65, but not NF-*κ*B-p50, is constitutively expressed in SKOV-3 and OVCAR-3 ovarian cancer cell lines. However, TOV-21G cells express endogenously both NF-*κ*B-p65 and NF-*κ*B-p50.

**Figure 6:**
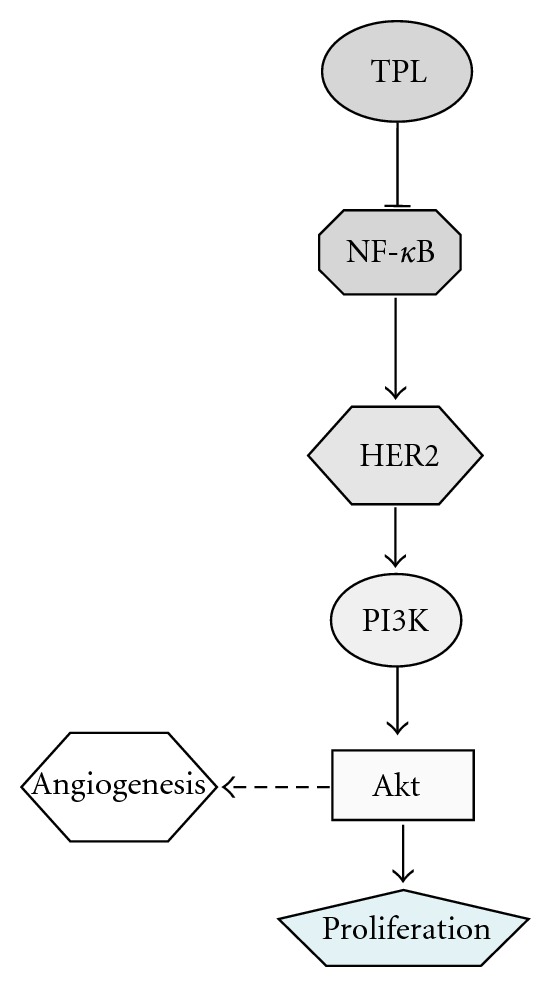
A schematic model demonstrating the effects of TPL on the regulation of NF-*κ*B and HER2. TPL inhibits tumor proliferation via downregulation of NF-*κ*B and HER2.

HER2 activation is often associated with invasion and proliferation through its downstream Ras/Raf/MAPK and PI3K/Akt signaling pathways [9, 10]. It is noteworthy that our results (Figures 2(c) and 2(d)) illustrate that in the HER2-overexpressing SKOV-3 (p53-null) cancer cells, PI3K/Akt signaling is dominantly activated as a result of increased HER2 activation and in the absence of p53, which has been reported to repress PI3K/Akt activity [20, 21]. In other words, although MAPK is also activated in ovarian carcinoma cells, it may not be the major pathway for TPL-mediated suppression of HER2 signaling in SKOV-3 cells (Figure 6).

 In LPS-stimulated macrophages, TPL downregulates the gene expression of COX-2, iNOS, and IL-1*β* [22]. Clinical trials using the extract of *T. wilfordii* or TPL have been reported. Wu and Guo studied the clinical effects of TPL in patients with psoriasis vulgaris and observed about 75% effectiveness [23, 24]. Tao et al. conducted a randomized double-blind study to assess the efficacy and tolerability of *T. wilfordii* root extracts in patients with rheumatoid arthritis (RA) and found therapeutic benefits in 60% of patients with high tolerability at the tested doses [24, 25]. In addition to immunosuppressive effects, TPL inhibits proliferation, angiogenesis, and invasion of a variety of cancer cells [2]. Our present work demonstrates that NF-*κ*B protein expression is inhibited by TPL treatment, which is consistent with most of the previous studies [11, 26, 27]. However, there is also evidence to suggest that TPL inhibits NF-*κ*B transcriptional activity by blocking the association of the p65 subunit with CREB-binding protein/p300 [11]. Thus, TPL may exert dual effects, through interactions with p65/CBP and by reducing p65 protein expression.

NF-*κ*B is a complex consisting of a heterodimer of p50 and p65 subunits. I*κ*B binds NF-*κ*B in the cytoplasm and prevents NF-*κ*B activation. The phosphorylation, ubiquitination, and degradation of I*κ*B lead to the release of the p50-p65 heterodimer, which translocates into the nucleus and then associates with the downstream promoter regions of multiple target genes, including vascular endothelial growth factor (VEGF) [28]. Angiogenesis typically occurs in RA, retinopathy, and tumor growth, as they are collectively called “angiogenic diseases” [29]. Thus, drugs used to treat RA and other angiogenic diseases may possess antitumor activity through antiangiogenic effects. VEGF has been identified as the most potent inducer of angiogenesis. Huang et al. demonstrated a concordant increase in NF-*κ*B activity with elevated VEGF mRNA in ovarian cancer cells, suggesting that the regulation of VEGF by NF-*κ*B is mediated at the transcriptional level [30]. We found that NF-*κ*B induces HER2 gene expression and subsequently stimulates Akt activity (data not shown). Akt has been implicated in the regulation of VEGF, which is activated by the mTOR/p70S6K pathway. mTOR is a 289-kDa phosphoinositide kinase-related serine/threonine kinase [31]. Through the formation of multimolecular complexes, rictor or raptor, the evolutionarily conserved TOR pathway controls many fundamental cellular functions, such as translation initiation, protein stability, transcription of ribosome and stress response genes, ribosomal biogenesis, and transfer RNA synthesis, and thereby plays a central role in the regulation of cell growth, proliferation, and survival [32, 33]. Therefore, we assume that TPL can inhibit angiogenesis through suppression of VEGF. Indeed, He et al. demonstrated that *T. wilfordii* and its terpenoids (TPL) possess antiangiogenic activity [34], which is consistent with our hypothesis.

Increasing evidence indicates that a small subpopulation of tumor cells, called tumor-initiating cells (TIC) or cancer stem cells, is not only responsible for the generation of the phenotypically diverse tumor cell populations but is also capable of long-term self-renewal, thereby supporting the growth and dissemination of cancers [35]. Several recent studies report that HER2 regulates TIC sphere formation and tumorigenicity [35–37]. Moreover, Notch, an important receptor in normal and malignant stem cells, may bind and regulate the HER2 promoter [37]. Thus, HER2 may become a superior target for TIC chemotherapy. Another way to identify putative TIC is through incubation with Hoechst dye and analysis by flow cytometry; a small population of cells that does not accumulate dye is called a side population [38]. The side population phenotype is mediated by the ATP-binding cassette (ABC) transporter protein family. These transporters are also called multidrug-resistant (MDR) ABC transporters. We previously demonstrated that TPL downregulates MDR protein expression and promotes apoptosis in the MDR-overexpressing KB oral cancer cell line [6]. In this study, we found that TPL decreases HER2 efficiently, implying that TPL may suppress TIC self-renewal via downregulation of HER2, but this remains to be investigated.
